# A Rare Case of Severe Folate Deficiency-Induced Pancytopenia

**DOI:** 10.7759/cureus.65858

**Published:** 2024-07-31

**Authors:** Adnan Abdullah, Ehsan Ul Shakoor, Shoayeb Sarwar, Tasnuba Raisa Jamil, Anum Hina

**Affiliations:** 1 Acute Medicine, Queen's Hospital, Barking, Havering, and Redbridge University Hospitals, London, GBR; 2 Acute Frailty and Medicine, King George Hospital, Barking, Havering, and Redbridge University Hospital Trust, London, GBR; 3 Internal Medicine, Sahiwal Medical College, Sahiwal, PAK

**Keywords:** alcohol misuse, folate deficiency, non-immune hemolytic anemia, anemia, “pancytopenia”

## Abstract

Chronic alcoholism is a well-documented cause of folate deficiency, with past studies revealing high prevalence rates among alcoholics. Despite mandatory folate fortification in the UK from 2021, individuals with chronic alcohol consumption remain susceptible to severe folate deficiencies. This case study explores the hematological impact of severe folate deficiency in a 38-year-old female chronic alcoholic who presented with pancytopenia. The patient’s symptoms included cough, shortness of breath, lethargy, reduced appetite, constipation, and rectal bleeding. Her medical history included polycystic ovarian syndrome and fatty liver disease. Blood tests revealed macrocytosis, pancytopenia, elevated bilirubin, and low serum folate levels. Management involved transfusions with packed red blood cells and oral folate supplementation, resulting in rapid hematological improvement. This case underscores the importance of early diagnosis and intervention for folate deficiency, particularly among chronic alcoholics.

Folate, or vitamin B9, is essential for DNA synthesis and red blood cell production. Chronic alcohol consumption disrupts folate metabolism and absorption, leading to deficiencies. The patient's improvement with folate supplementation highlights the efficacy of this treatment. This case emphasizes the need for ongoing monitoring and support for chronic alcoholics to prevent recurrent folate deficiency. Further studies are necessary to assess the long-term efficacy of folate-fortification programs and ensure they meet the needs of vulnerable groups, including those with chronic alcohol dependence.

## Introduction

Chronic alcoholism is known to cause folate deficiency. In a study, 80% of 70 chronic alcoholics admitted to a large United States of America (USA) urban hospital had low serum folate levels. Among them, 44% had severely deficient folate levels [1]. However, the study was conducted over 50 years ago in the United States. According to Public Health England, the prevalence of folate insufficiency, defined as serum folate levels below the World Health Organization's clinical threshold for deficiency of 7 nanomol/L, is approximately 5% or less among adults and children. However, this survey was conducted nine years ago [2]. Since the United Kingdom implemented mandatory fortification of food with folic acid in September 2021, the aim has been to increase folate levels in women of childbearing age, thereby reducing the incidence of folate deficiency and lowering the occurrence of neural tube defects such as spina bifida and anencephaly [3].

Folic acid, also known as vitamin B9, is an essential water-soluble nutrient. It plays a crucial role in human growth and development. Interestingly, patients with alcoholism often experience folic acid deficiency, which is a common nutritional issue. [4] Two separate clinical studies found that chronic alcoholics admitted to hospital emergency rooms for alcohol withdrawal had decreased intestinal absorption of 3H-labeled folic acid. Surprisingly, acute ethanol ingestion did not affect folate absorption. Chronic alcohol consumption can disrupt hepatic folate metabolism, affecting basolateral membrane transport, intrahepatic processing, and folate redistribution. This alteration leads to abnormal liver uptake and reduced folate storage [5,6].

Our case involved a chronic alcoholic patient with a severe isolated folate deficiency, while vitamin B12 levels remained normal. This deficiency led to pancytopenia. Although there have been a few case reports of pancytopenia due to isolated folate deficiency, the most recent report dates to 2010 [7].

## Case presentation

A 38-year-old female of Northern European descent arrived at the Accident and Emergency Department by ambulance. She presented with a one-week history of cough, shortness of breath, lethargy, and reduced appetite. Additionally, she had been experiencing constipation and rectal bleeding for the past three months, which had been previously diagnosed as an anal fissure by her general practitioner (GP). To address her cough and shortness of breath, she purchased oral amoxicillin from a local pharmacy. While in the emergency department, she vomited a couple of times, accompanied by dizziness and central chest pain. She denied fever, abdominal pain, falls, weight loss, and hematuria, and there was no recent travel history. Her medical history included polycystic ovarian syndrome, diagnosed in September 2013, and fatty liver disease, diagnosed in 2014. The patient is a heavy smoker (one pack per day) and has been consuming alcohol for the past 15 years. In the three days leading up to admission, she had been drinking more than 30 units of vodka daily, although she mentioned being alcohol-free for the preceding three months. She denied intravenous drug use. 

In an accident and emergency, she was seen by the general surgery team, and per rectal examination was performed. She had a hard stool in the rectum with no bleeding and a preserved anal tone. Following a surgical review, she was referred to the medicine team. 

Upon admission, the patient’s vital signs were as follows: blood pressure of 93/66 mm Hg, heart rate of 97 beats per minute, oxygen saturation of 97% on room air, respiratory rate of 18 breaths per minute, and a body temperature of 37 °C. She was mildly icteric and had a Glasgow Coma Scale score of 15 out of 15.

Table 1 displays the patient’s blood test results at admission, which revealed macrocytosis with pancytopenia, elevated bilirubin levels, increased C-reactive protein, and elevated creatinine with a low estimated glomerular filtration rate (eGFR).

**Table 1:** Routine blood test done at the time of admission.

Parameter	Result	Reference Range
White cell count	3.7	3.8–11.0 × 10^9^/L
Haemoglobin (Hb)	60	115–155 g/L
Mean cell volume: red cell (MCV)	105.8	80.0–96.0 fL
Platelet count (Plt)	67	150–400 × 10^9^/L
Neutrophils	2.0	2.0–7.5 × 10^9^/L
Lymphocytes	1.6	1.5–4.0 × 10^9^/L
Eosinophils	0.1	0.0–0.4 × 10^9^/L
Immature granulocytes	0.51	0.0–0.5 × 10^9^/L
Nucleated red blood cells %	2.40%	
Reticulocytes (%)	1.5%	0.2–2.0%
Reticulocytes (conc)	23.20	50–100 × 10^9^/L
Prothrombin time (PT)	13.1	9–13 s
Activated partial thrombin time (APTT)	26.7	20–33 s
Thrombin time	16.9	13–17 s
Sodium	136	133–146 mmol/L
Potassium	3.6	3.5–5.3 mmol/L
Urea	5.2	2.5–7.9 mmol/L
Creatinine	112	46–84 μmol/L
Estimated glomerular filtration rate (eGFR)/1.73 m²	54	ML/min
Total protein	62	60–80 g/L
Albumin	36	35–50 g/L
Alkaline phosphatase (ALP)	103	30–130 U/L
Alanine transaminase (ALT)	26	<33 U/L
Total bilirubin	95	1–21 μmol/L
Adjusted calcium	2.36	2.20–2.60 mmol/L
Phosphate	1.08	0.8–1.5 mmol/L
C-reactive protein	21	<5 mg/L

**Figure 1:**
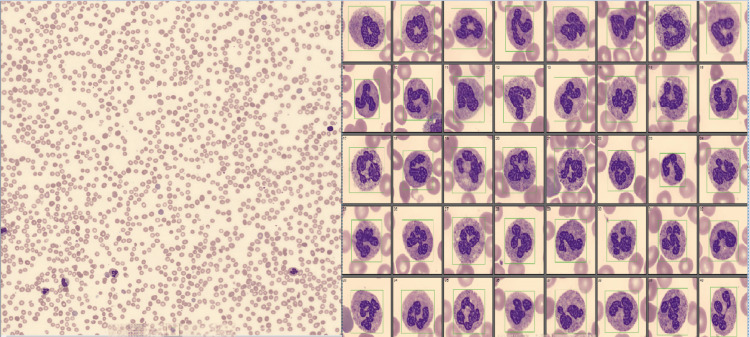
Microscopic appearance of pancytopenia and hyper segmented neutrophils on blood film taken on the day of admission.

The blood film showed macrocytosis of the red cells with polychromasia, teardrop poikilocytosis, target cells, and occasional irregularly contracted cells. The blood film also noted true thrombocytopenia: no clumps, no fragments. Hyper-segmented neutrophils and toxic granulation were also observed. The patient also had a chest X-ray, which was unremarkable. Figure 1 shows the blood film depicting pancytopenia and hyper-segmented neutrophils.

**Figure 2:**
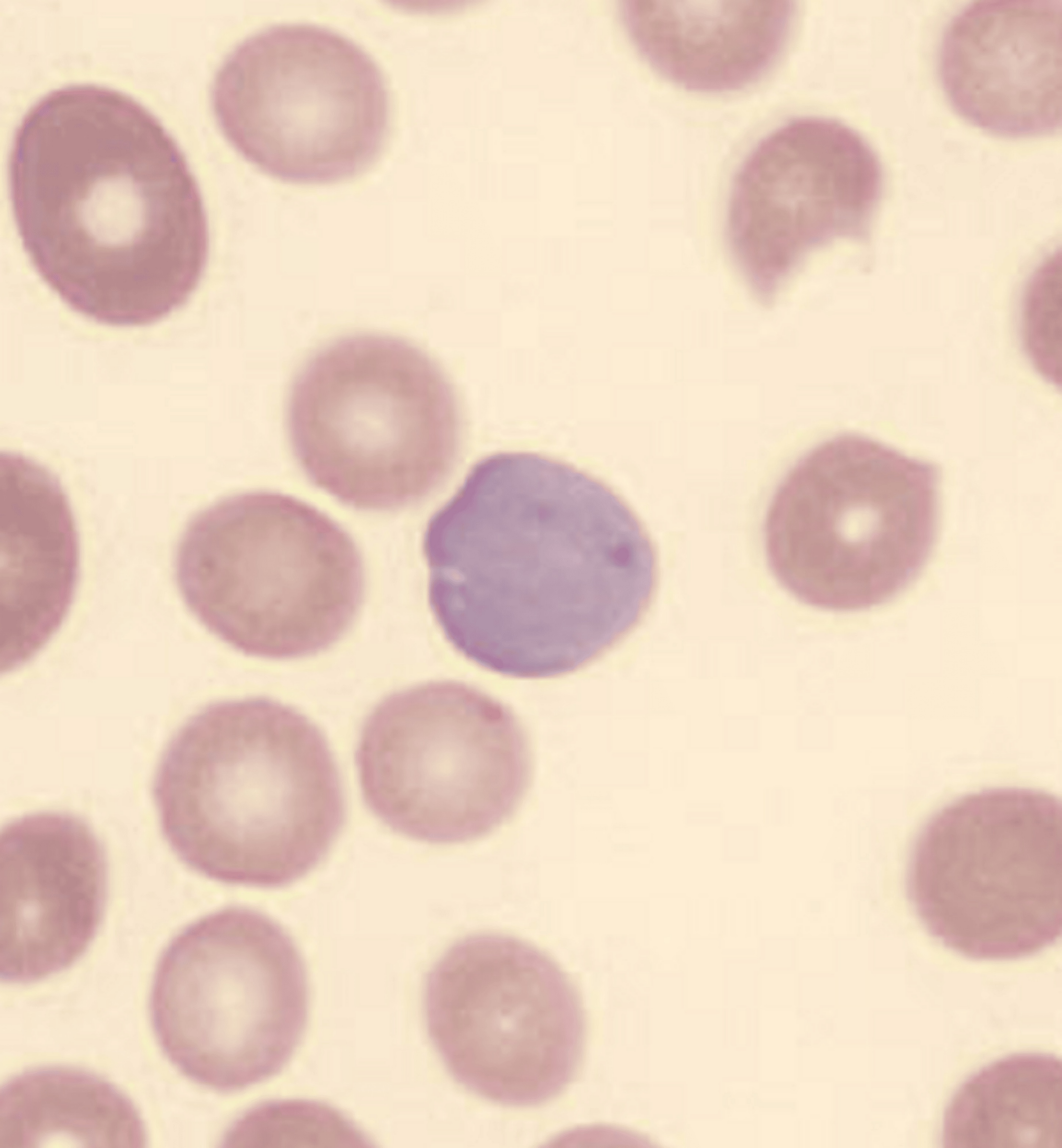
Basophilic stippling seen in the blood film in context of macrocytosis on blood film at the time of admission.

Figure 2 shows basophilic stippling in the blood film, which refers to the presence of numerous basophilic granules dispersed throughout the cytoplasm of erythrocytes in a peripheral blood smear [8].

After reviewing the routine blood tests, the patient was transfused with three units of packed red blood cells and was referred to the hematology team for further advice. According to the hematology team’s recommendation, the patient underwent testing for vitamin B12, folate levels, iron profile, LDH (lactate dehydrogenase), GGT (gamma-glutamyl transferase), immunoglobulins, connective tissue antibodies, haptoglobin, and the DAT (direct agglutination test) screen.

Table 2 presents a comprehensive overview of her blood work, encompassing the above-mentioned investigations.

**Table 2:** Comprehensive investigation done for her anemia after blood product transfusions.

Parameter	Result	Reference Range
Haemoglobin (Hb)	71 g/L	115–155 g/L
Mean corpuscular volume (MCV)	99.5 fL	80.0–96.0 fL
Red cell distribution width (RDW)	25.2%	9.0–15.0%
Platelets (PLT)	56 × 10^9^/L	150–400 × 10^9^/L
Neutrophils	1.7 × 10^9^/L	2.0–7.5 × 10^9^/L
Lymphocytes	1.2 × 10^9^/L	1.5–4.0 × 10^9^/L
Immature granulocytes	0.25 × 10^9^/L	0.0–0.5 × 10^9^/L
Nucleated red blood cells %	0.90%	
Reticulocytes (%)	2.2%	0.2–2.0%
Reticulocytes concentration	46.20	50–100 × 10^9^/L
Reticulocyte haemoglobin	43.20	28–34 pg
Prothrombin time (PT)	11.9 s	9–13 s
Activated partial thromboplastin time (APTT)	27.3 s	20–33 s
Thrombin time	16.1 s	13–17 s
Potassium	3.6 mmol/L	3.5–5.3 mmol/L
Urea	4.5 mmol/L	2.5–7.9 mmol/L
Creatinine	65 μmol/L	46–84 μmol/L
Estimated GFR (eGFR)	>90 mL/min	
Total protein	55 g/L	60–80 g/L
Albumin	32 g/L	35–50 g/L
Alkaline phosphatase (ALP)	95 U/L	30–130 U/L
Alanine transaminase (ALT)	22 U/L	<33 U/L
Aspartate transaminase (AST)	87 U/L	<32 U/L
Total bilirubin	79 μmol/L	1–21 μmol/L
Conjugated bilirubin	62 μmol/L	<6 μmol/L
Globulin	23 g/L	18–36 g/L
Adjusted calcium	2.28 mmol/L	2.20–2.60 mmol/L
Gamma glutamyl transferase (GGT)	154 U/L	<40 U/L
Lactate dehydrogenase (LDH)	>1800 IU/L	<250 mg/L
C-reactive protein	21 mg/L	<5 mg/L
Iron	7 μmol/L	5.8–34.8 μmol/L
Unsaturated iron binding capacity	39 μmol/L	
Iron saturation	15%	13–45%
Total Iron binding capacity	46 μmol/L	30–105 μmol/L
Alpha feto protein	4.1 kU/L	<5.8 kU/L
Serum IgG	12.10 g/L	5.5–16.5 g/L
Serum IgA	1.90 g/L	0.80–4.00 g/L
Serum IgM	0.35 g/L	0.50–1.90 g/L
Haptoglobin	<0.1 g/L	0.3–2.0 g/L
Ferritin	974.0 μg/L	30–400 g/L
Vitamin B12	201 ng/L	191–663 ng/L
Serum folate	<0.6 μg/L	3.9–26.8 μg/L
		Replete >5.3 μg/L
		Indeterminate: 3.9–5.3 μg/L
		Deficient: <3.9 μg/L
ANA (tissue block)	Negative	
Gastric parietal cell	Negative	
Mitochondrial	Negative	
Anti-smooth muscle abs	Negative	
Liver/kidney micro.	Negative	

A direct agglutination test came back negative. The patient was also negative for human immunodeficiency virus (HIV), hepatitis B & C, and cytomegalovirus.

**Figure 3:**
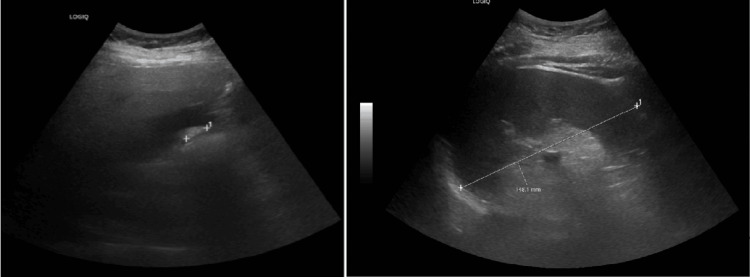
Ultrasound abdomen showing echogenic sludge noted within the gallbladder lumen (left) and enlarged spleen (right).

Reviewing the abnormal liver function test, ultrasonography (USG) of the abdomen was also requested, which showed that the liver appeared enlarged with a regular outline and bright parenchyma, suggestive of diffuse fatty infiltration. No focal lesions were seen. The gallbladder had a regular outline, with echogenic sludge and a 1.7 cm echogenic focus, possibly indicative of a gallstone (Figure 3). The common bile duct (CBD) measured 6 mm and showed no intrahepatic bile duct dilatation. The spleen was enlarged (14.8 cm in longitudinal axis), as shown in Figure 3, while both kidneys appeared normal in size and shape, with no calculi or hydronephrosis.

Treatment

After the investigations mentioned above, the hematology team concluded that the patient’s pancytopenia was likely due to severe folate deficiency. Consequently, the patient was discharged with a three-month supply of folate (a tablet of folic acid, 5 mg, once a day) and scheduled for repeat blood tests after two weeks at the ambulatory clinic. Lifelong folate supplementation may be required if the patient continues to drink alcohol. There were no further episodes of rectal bleeding, and the gastroenterology team arranged outpatient follow-up. Additionally, the patient received support from the alcohol liaison team during admission and continued follow-up through the Change Grow Live (CGL) service in the community.

Follow up

The patient attended the follow-up in the ambulatory clinic after two weeks, and the blood test showed dramatic improvement. Table 3 shows the dramatic improvement made after two weeks of folate supplementation.

**Table 3:** Routine blood tests done in the ambulatory clinic follow up.

Parameter	Result	Reference range
White cell count	8.2 × 10^9^/L	3.8 –11.0 × 10^9^/L
Haemoglobin (Hb)	105 g/L	115–155 g/L
Red cell count (RBC)	3.36 × 10^12^/L	3.80–5.50 × 10^12^/L
Haematocrit	0.33	0.37–0.47
Mean cell volume: red cell (MCV)	97.9 fL	80.0–96.0 fL
Mean cell haemoglobin (MCH)	31.3 pg	27.0–32.0 pg
Mean corpuscular hemoglobin concentration	319 g/L	320–360 g/L
Red cell distribution width	17.7%	9.0–15.0%
Platelet count (Plt)	382 × 10^9^/L	150–400 × 10^9^/L
Platelet distribution width (PDW)	11.4%	
Mean platelet volume	10.6 fL	
Neutrophils	4.9 × 10^9^/L	2.0–7.5 × 10^9^/L
Lymphocytes	2.3 × 10^9^/L	1.5–4.0 × 10^9^/L
Monocytes	0.7 × 10^9^/L	0.2–0.8 × 10^9^/L
Eosinophils	0.2 × 10^9^/L	0.0–0.4 × 10^9^/L
Basophils	0.1 × 10^9^/L	0.0–0.1 × 10^9^/L
Immature granulocytes	0.07 × 10^9^/L	0.0–0.5 × 10^9^/L
Nucleated red blood cells (%)	0.00%	
Prothrombin time (PT)	11.4 s	9–13 s
Activated partial thrombin time (APTT)	28.7 s	20–33 s
Thrombin time	17.1 s	13–17 s
Sodium	141 mmol/L	133–146 mmol/L
Potassium	4.4 mmol/L	3.5–5.3 mmol/L
Urea	2.2 mmol/L	2.5–7.9 mmol/L
Creatinine	49 μmol/L	46–84 μmol/L
Estimated glomerular filtration rate (eGFR)/1.73 m²	>90 mL/min	
Total protein	67 g/L	60–80 g/L
Albumin	36 g/L	35–50 g/L
Alkaline phosphatase (ALP)	109 U/L	30–130 U/L
Alanine transaminase (ALT)	22 U/L	<33 U/L
Total bilirubin	36 μmol/L	1–21 μmol/L
Globulin	31 g/L	18–36 g/L
Calcium	2.26 mmol/L	
Adjusted calcium	2.41 mmol/L	2.20–2.60 mmol/L
Phosphate	1.00 mmol/L	0.8–1.5 mmol/L
Magnesium	0.73 mmol/L	0.7–1.0 mmol/L

As the patient made good improvements, she was discharged from the ambulatory clinic for follow-up with her GP.

## Discussion

Common causes of pancytopenia include decreased production due to nutritional deficiencies or bone marrow failure, such as aplastic anemia. This condition can be idiopathic or result from infections, drug toxicity, chemotherapy, inadequate intake, or malabsorption. Pancytopenia also results from bone marrow infiltration by malignancies or granulomatous disorders or from the peripheral destruction of cells due to autoimmune conditions or splenic sequestration. During the COVID-19 pandemic, pancytopenia has been reported as a complication of SARS-CoV-2, with bone marrow aspiration showing viral infection and infiltration [9].

This case of a 38-year-old female presenting with severe hematological abnormalities highlights the critical impact of chronic alcoholism and folate deficiency on health. The patient's initial presentation included a one-week history of cough, shortness of breath, lethargy, and reduced appetite, compounded by a three-month history of constipation and rectal bleeding, previously diagnosed as an anal fissure. These symptoms, alongside her recent alcohol binge, led to a complex clinical picture requiring urgent medical intervention. The patient's history of heavy smoking and prolonged alcohol consumption exacerbated her condition, leading to severe folate deficiency, as evidenced by her macrocytosis, pancytopenia, elevated bilirubin, and low serum folate levels.

Folate deficiency is known to result in a diminished production of purines and pyrimidines, which subsequently impacts DNA production and cell division. This effect is particularly evident in reducing red blood cells, leading to anemia [10]. Folate-deficiency-induced pancytopenia is quite rare [7]. In instances where folate deficiency is suspected, the diagnosis can typically be confirmed through blood tests without needing a bone marrow biopsy [11]. If serum folate levels are inconclusive, the total homocysteine level can be checked, as it tends to be elevated in the presence of folate deficiency [12]. 

Standard treatment for isolated folate deficiency usually involves oral folate supplementation over three to four months, with subsequent follow-ups [13]. This patient also exhibited signs of non-immune-mediated hemolysis, likely intramedullary, as indicated by increased LDH and bilirubin levels, decreased haptoglobin, and a negative direct agglutination test [14]. This condition frequently occurs with combined deficiencies of vitamin B12 and folate; however, in our case, there was an isolated folate deficiency [15]. Such cases on the extreme spectrum of severe deficiency are extremely rare in developed countries where folate fortification is prevalent. In the UK, a severe folate deficiency case was last reported in 2010 with pancytopenia, and there was no report of non-immune-mediated hemolysis [7]. This was before folate fortification. In the UK, no further population studies have been conducted to assess the efficacy of the folate fortification program after its implementation [2].

## Conclusions

This case highlights the significant impact of severe folate deficiency on hematological health, particularly in the context of chronic alcoholism. The patient presented with symptoms indicative of pancytopenia and severe folate deficiency, which were effectively managed through folate supplementation. The patient's rapid improvement following treatment underscores the importance of early diagnosis and intervention.

Historically, chronic alcoholism has been closely associated with folate deficiency, as evidenced by studies showing high prevalence rates among alcoholics. This condition disrupts folate metabolism and absorption, leading to hematological abnormalities such as pancytopenia. Despite mandatory folate fortification in foods in the UK since 2021, this case demonstrates that individuals with chronic alcohol consumption can still experience severe folate deficiencies. The patient's clinical presentation, characterized by macrocytosis, elevated bilirubin, and low serum folate, aligns with the known pathophysiological effects of folate deficiency on DNA synthesis and red blood cell production. Successful management with oral folate supplementation, resulting in significant hematological improvement, highlights the efficacy of this standard treatment.
